# Diversity of Growth Patterns Probed in Live Cyanobacterial Cells Using a Fluorescent Analog of a Peptidoglycan Precursor

**DOI:** 10.3389/fmicb.2018.00791

**Published:** 2018-04-24

**Authors:** Ju-Yuan Zhang, Gui-Ming Lin, Wei-Yue Xing, Cheng-Cai Zhang

**Affiliations:** ^1^Key Laboratory of Algal Biology, Institute of Hydrobiology, Chinese Academy of Sciences, Wuhan, China; ^2^University of Chinese Academy of Sciences, Beijing, China

**Keywords:** cyanobacteria, peptidoglycan, growth pattern, cell wall, heterocyst, HADA

## Abstract

Cyanobacteria were the first oxygenic photosynthetic organisms during evolution and were ancestors of plastids. Cyanobacterial cells exhibit an extraordinary diversity in their size and shape, and bacterial cell morphology largely depends on the synthesis and the dynamics of the peptidoglycan (PG) layer. Here, we used a fluorescence analog of the PG synthesis precursor D-Ala, 7-Hydroxycoumarin-amino-D-alanine (HADA), to probe the PG synthesis pattern in live cells of cyanobacteria with different morphology. They displayed diverse synthesis patterns, with some strains showing an intensive HADA incorporation at the septal region, whereas others gave an HADA signal distributed around the cells. Growth zones covering several cells at the tips of the filament were present in some filamentous strains such as in *Arthrospira*. In *Anabaena* PCC 7120, which is capable of differentiating heterocysts for N_2_ fixation, PG synthesis followed the cell division cycle. In addition, an HADA incorporation was strongly activated from 12 to 15 h following the initiation of heterocyst development, indicating a thickening of the PG layer in heterocysts. The PG synthesis pattern is diverse in cyanobacteria and responds to developmental regulation. The use of fluorescent analogs may serve as a useful tool for understanding the mechanisms of cell growth and morphogenesis operating in these organisms.

## Introduction

Cyanobacteria are the only prokaryotes capable of oxygenic photosynthesis. Their occurrence on the Earth dates back to at least 2.45–2.32 Ga, but they may have first appeared even earlier, as evidenced by the fossils records (Bekker et al., 2004; Jeltsch, 2013). Cyanobacteria were responsible for the rise of oxygen levels, and certain ancient freshwater cyanobacteria were thought to be the ancestors of plastids through endosymbiosis with a heterotrophic host (Ponce-Toledo et al., 2017). During the long history of evolution, present-day cyanobacteria display a high degree of diversity in terms of morphology, living in the form of unicellular or filamentous strains, or in colonies. Depending on the strains, cyanobacterial cells also show a high diversity in their shape (round, spiral or rod), with sizes ranging from approximately 0.5 μm in the case of marine *Prochlorococcus* to as large as 3–5 μm for freshwater *Anabaena*/*Nostoc* strains. How to account for this morphological diversity among cyanobacteria remains unclear compared to the understanding of other model organisms such as *Escherichia coli* and *Bacillus subtilis*.

One major element that contributes to the size and cell morphology in bacteria is the peptidoglycan (PG) layer (for a review, see Randich and Brun, 2015). The PG layer is a macromolecule that surrounds bacterial cells and maintains cell shape. It is composed of a polymer network of N-acetylglucosamine and N-acetylmuramic acid cross-linked with pentapeptide chains. Such networks are so rigid that isolated PG molecules (sacculi) are able to retain the shape of their original cells. Although rigid, the PG layer is highly dynamic and is constantly remodeled in order to keep pace with cell growth, division, or morphogenesis. Multiple modes of cell growth have been described in bacteria: lateral elongation, septation accompanying the process of cell constriction during cell division, polar elongation at the cell poles, or medial elongation emanating from the growth at the division plane (Randich and Brun, 2015). In one bacterium, such as *E. coli*, multiple modes of growth may together contribute to cell growth and morphogenesis. Two molecular machineries direct the synthesis of PG during cell cycle: the elongasome, responsible for insertion of new PG along the sidewall, and the divisome, which is located at the cell division plane. FtsZ treadmilling directs the dynamic synthesis of PG during cell constriction, whereas MreB coordinates the activity of the elongasome (Bisson-Filho et al., 2017; Yang et al., 2017). Both MreB and FtsZ are cytoskeletal elements (Busiek and Margolin, 2015) and are conserved in cyanobacteria (Zhang et al., 1995; Sakr et al., 2006; Hu et al., 2007).

Although the pentapeptides linking the sugar chains in PG may vary in composition depending on the bacteria, they usually contain D-Ala at the fourth and fifth positions. A fluorescent derivative of vancomycin (Van-FL), which tightly binds to the D-Ala-D-Ala dipeptide, has been used to trace PG biosynthesis sites and cell growth in a spectrum of bacteria (Tiyanont et al., 2006; Divakaruni et al., 2007; Lehner et al., 2013; Mariscal et al., 2016). Recently, several fluorescent D-amino acids (FDAAs) have been developed to probe the growth mode in bacterial cells by revealing the active part in a cell where PG synthesis occurs (Kuru et al., 2015). Van-FL is an analog of an antibiotic, whereas FDAAs, as analogs of native D-amino acids, can be incorporated into pentapeptide chains during PG synthesis with less influence on cell growth. Therefore, FDAAs can be used to trace PG synthesis in live cells. In Gram-negative prokaryotes, including cyanobacteria, the PG layer is within the periplasmic space, between the inner and outer membranes. In general, Gram-negative prokaryotes have a thinner PG layer compared to Gram-positive prokaryotes; however, cyanobacteria have a PG layer that is more reminiscent of those typically found in Gram-positive bacteria, such that it is thicker and has a higher degree of cross-linking (Hoiczyk and Hansel, 2000). Since little is known about the growth mode in cyanobacteria, we used one D-Ala analog, 7-hydroxycoumarin-3-carboxylic acid–D-alanine (HADA), to probe the growth mode in cyanobacteria with different morphologies. HADA emits blue fluorescence, with little interference from the bright red fluorescence from the photosynthetic pigments. Our results reveal a great diversity in the mode of cell growth among cyanobacteria. We observed certain growth patterns that appeared to be unique. For example, the PG synthesis zone covered several cells at the tip of the filaments in some filamentous cyanobacteria. We also characterized the activity of PG synthesis in the heterocyst-forming cyanobacterium *Anabaena* PCC 7120 (also known as *Nostoc* PCC 7120), as an example of PG-layer remodeling during cell differentiation (Nicolaisen et al., 2009). Heterocysts, which are specialized in N_2_ fixation, are induced upon combined nitrogen starvation (Zhang et al., 2006; Herrero et al., 2016). Several genes encoding enzymes involved in PG metabolism have already been shown to be required for heterocyst development or functioning (Lázaro et al., 2001; Zhu et al., 2001; Lehner et al., 2011; Berendt et al., 2012; Videau et al., 2016; Bornikoel et al., 2017; Zheng et al., 2017). Our results showed an increased PG synthesis activity during heterocyst maturation, after the deposition of the polysaccharide layer, which led to a thick layer of PG surrounding the mature heterocyst. These results increase our understanding of the molecular mechanisms underlying PG synthesis and cellular morphogenesis in cyanobacteria.

## Materials and Methods

### Reagents

Synthesis of HADA was carried out according to a previously described protocol (Kuru et al., 2015). HADA stock solution was prepared in DMSO at a concentration of 100 mM and stored at -20°C before use. Aztreonam was purchased from Sigma-Aldrich (Cat: PHR1785).

### Cyanobacterial Strains and Growth Conditions

*Anabaena* PCC 7120 and *Synechocystis* PCC 6803 were maintained in our lab; *Synechococcu*s *elongatus* PCC 7942, *Arthrospira* sp. FACHB 792, *Spirulina subsalsa, Microcystis* PCC 7806, and *Oscillatoria animalis* were obtained from Freshwater Algae Culture Collection at the Institute of Hydrobiology (FACHB); and *Leptolyngbya* sp. CB006, isolated from Tigris River in Baghdad City, was kindly provided by Ibrahim J. Abed in Baghdad University.

*Arthrospira* and *Spirulina* were cultivated in the *Arthrospira* medium described previously (Aiba and Ogawa, 1977). *Anabaena*, *Oscillatoria, Leptolyngbya*, *Synechocystis*, and *Synechococcu*s *elongtus* were grown in BG11 medium (Stanier et al., 1971). *Microcystis* was grown in BG11_0_ medium (BG11 without combined nitrogen) supplemented with 2 mM NaNO_3_ and 10 mM NaHCO_3_. All strains were grown axenically at 30°C in an incubator with the orbital shaking speed of 180 rpm and the light density of 30 μmol m^-2^s^-1^.

To measure the growth of cyanobacterial strains, the fresh culture of each strain was inoculated into three 250-ml flasks with each flask containing 30 ml of medium, to an initial optical density of 0.05 at 750 nm (OD_750_), and grown under conditions described above. The OD_750_ of each culture was measured every 12 h until the OD was 1.0. The growth curves were plotted using the 2-based logarithm of the OD_750_ reads (Y-axis) and sampling time (X-axis). The growth rate (μ) of each strain was calculated from the slope of the linear region (corresponding to the exponential growth) in the semilogarithmic curve. The generation time, or doubling time (d), was calculated using the equation: *d* = 1/μ. The generation times of the strains were as follows: *Synechocystis*, *Microcystis* and *S. elongatus*, approximately 17 h; *Oscillatoria*, 28 h; *Spirulina*, 11 h; *Arthrospira*, 25 h; *Leptolyngbya*, 13 h; *Anabaena*, 20 h.

### Construction of Plasmids and Strain

The sequences of all the primers used in this study are listed in Supplementary Table S1. All the sequences of the plasmids used in this study have been deposited into GenBank.

The vector pCint2 (GenBank accession number: MH050934; Supplementary Figure S1) is a derivate of pRL271 (Cai and Wolk, 1990) with the erythromycin-resistant gene cassette deleted from the original plasmid. To construct pCint2, the vector pRL271 was amplified with the primers of PCINT2a and PCINT2b and the 5450 bp PCR fragment was circularized using the Vazyme ClonExpress II One Step Cloning Kit (Cat: C112-01). A sequence encoding a protein linker, a cyanobacterial codon-optimized *syfp2* ORF, a TwinStrep tag, and an artificial transcriptional terminator was synthesized by GenScript and cloned into the vector pUC57-simple (GenScript) via EcoRV, resulting in the plasmid pSYFP2 (GenBank accession number: MH050935; Supplementary Figure S2). To make pSYFP2-sp (GenBank accession number: MH050936; Supplementary Figure S3), a spectinomycin/streptomycin resistance gene (1053 bp) was amplified from the omega fragment (Prentki and Krisch, 1984) using the primers of PspF214m and PspR797, and cloned into ApaI-linearized pSYFP2 using the Vazyme ClonExpress II One Step Cloning Kit. The plasmid pFtsZ-SYFP2 was constructed as follows. Two *ftsZ* regions were amplified from the chromosome of *Anabaena* PCC 7120 with the primer pairs of Palr3858F160/Palr3858R1284 (product size: 1167 bp) and Palr3858F1353/Palr3858R2377 (product size: 1062 bp), respectively. The YFP coding region together with the spectinomycin/streptomycin resistance gene (2124 bp) was amplified from the plasmid pSYFP2-sp with the primers of Psyfp2spF and Psyfp2spR. The three PCR fragments were inserted into PstI/Xho linearized pCint2 using the Vazyme ClonExpress MultiS One Step Cloning Kit (Cat: C113-01), resulting in pFtsZ-SYFP2 (GenBank accession number: MH050937; Supplementary Figure S4). All plasmids were verified by sequencing.

The plasmid pFtsZ-SYFP2 was transferred into wildtype *Anabaena* PCC7120 by conjugation to make the *Anabaena* FtsZ-SYFP translational fusion strain. The methods of conjugation and selection of desired colonies followed a previously described procedure (Cai and Wolk, 1990; Elhai et al., 1997). The genotype of the obtained strain was verified by PCR (data not shown). The translational fusion strain was grown in BG11 medium supplemented with 5 μg ml^-1^ spectinomycin and 2.5 μg ml^-1^ streptomycin.

### HADA Labeling

For pulse labeling, exponentially growing cells were diluted to OD_750_ = 0.2 in a medium containing 800 μM HADA and grown for 2∼10% generation time, then washed with cold 1× phosphate buffered saline (PBS, which has a pH of 7.4 and contains 137 mM NaCl, 2.7 mM KCl, 8 mM Na_2_HPO_4_, and 14.6 mM KH_2_PO_4_) and imaged immediately.

For longer labeling times, exponentially growing cells were diluted to OD_750_ = 0.2 in media containing 200 μM HADA and grown for 1∼2 generation times. For heterocyst labeling, 200 μM HADA was added into BG11_0_ medium at different time points. HADA-labeled cells were washed either with the growth medium to remove free HADA from the culture and allowed to grow without HADA, or with cold 1× PBS and imaged immediately.

For HADA labeling after cell division inhibition, the FtsZ-YFP translational fusion strain exponentially growing in BG11 was treated with 100 μM aztreonam for 36 h, followed by the combined treatment of 100 μM aztreonam and 200 μM HADA for 24 h.

### Microscopy and Image Processing

HADA has fluorescence properties similar to blue fluorescent proteins (BFPs), with a maximal excitation wavelength at 405 nm and the maximal emission at 460 nm (Kuru et al., 2015). The Nikon filter sets of BFP (EX379-401, DM420LP, EM435-485), B-2A (EX450-490, DM515LP, EM515LP), and YFP HYQ (EX490-510, DM520-580, EM520-550) were used to image HADA fluorescence, the fluorescence of photosynthetic pigments, and FtsZ-YFP fluorescence, respectively. All microscopic images were taken using a Nikon Ti-E inverted fluorescence microscope equipped with a Plan Apo 100×/1.70 Oil Ph3 DM objective lens, an HQ2 CCD camera. All HADA pictures were taken with the same exposure time (1 s). Merging of image channels, straightening of spiral or curved cyanobacterial filaments, and image quantification were performed with ImageJ 1.50 (Schneider et al., 2012).

## Results

### Cell Growth of Unicellular Strains Depends on Major Septal and Minor Peripheral PG Synthesis (*Synechocystis* and *S. elongatus*) or Medial Elongation (*Microcystis*)

We tested the effect of different HADA concentrations on the growth of strains used in this study and found that a concentration of 200 μM had little effect, whereas a concentration up to 400 or 800 μM slightly impeded cell growth, making cells yellowish (Supplementary Figure S5). Thus, we used 200 μM for long-term labeling and 800 μM for pulse labeling for half an hour or one hour, following the rules that pulse-labeling times correspond to 2–5% of a generation time, and long-term labeling times correspond to 1–2 generation times (Kuru et al., 2015). The doubling time of the strains used in this study varied between 11 and 28 h under our culture conditions.

**FIGURE 1:**
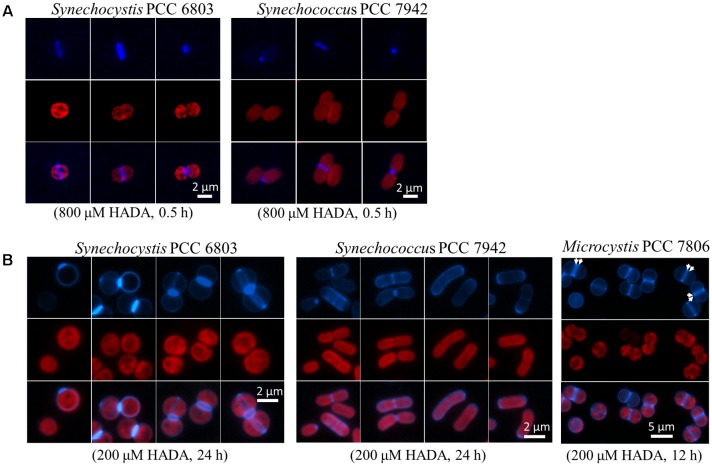
HADA labeling in three unicellular cyanobacteria: *Synechocystis* PCC 6803, *Synechococcus elongatus* PCC 7942, and *Microcystis* PCC 7806. **(A)** Pulse labeling of *Synechocystis* and *Synechococcus elongatus* with a high concentration of HADA (800 μM) for 30 min, followed by removal of HADA by washing and observation by fluorescence microscopy. **(B)** Continuous labeling with a low concentration of HADA in *Synechocystis*, *Synechococcus elongatus*, and *Microcystis*. For *Synechocystis* and *Synechococcus elongatus*, three or four sets of photos are shown, and each demonstrates a representative HADA labeling pattern. HADA signal is in blue, and photosynthetic pigment in red, and the overlay for the images of same cells acquired through the two channels. Arrows indicates the pre-septal division mode in *Microcystis*.

We first tested the synthesis of the PG layer in two model unicellular strains, representing each a distinct cellular morphology of cyanobacteria. *Synechocystis* is round-shaped, whereas *S. elongatus* is rod-shaped. Both strains have a binary division mode, with a generation time of approximately 17 h under our culture condition. We first performed pulse labeling for 30 min with 800 μM HADA (**Figure 1A**). In both strains, intensive incorporation of HADA occurred at the mid site, starting from young cells before any sign of cell constriction, until the very end of the cell cycle when cell constriction was almost finished. Occasionally, HADA labeling at one cell pole could be observed, due to the separation of two daughter cells during the course of HADA pulse labeling. Thus, both strains, with different cell shapes, showed substantial incorporation of HADA at the septal region. We also performed continuous HADA labeling for up to 24 h with 200 μM HADA (**Figure 1B**). For both strains, in addition to intensive septal labeling, weaker peripheral HADA fluorescence could also be seen, indicating that these strains had a low peripheral PG synthesis activity. For *Synechocystis*, a new division plane positioned perpendicularly to the older one could often be found in one or both daughter cells before they separated. This finding is consistent with the division pattern of this strain (Marbouty et al., 2009).

We also studied the PG synthesis pattern in *Microcystis* PCC 7806 which is a round-shaped strain known for its production of the toxin microcystin (Dittmann and Wiegand, 2006). *Microcystis* and *Synechocystis* showed no obvious difference in HADA incorporation when pulse-labeled for 30 min with 800 μM HADA (data not shown). Like *Synechocystis*, *Microcystis* also showed a septal and peripheral HADA incorporation with continuous HADA labeling for 12 h at a concentration of 200 μM (**Figure 1B**). Interestingly, a labeling pattern of double stripe at the mid cell position could be found in many cells (indicated by arrows in **Figure 1B**). Such a growth pattern has been reported in some bacteria, called pre-septal or medial elongation, corresponding to PG synthesis bordering the division plane before full assembly of the divisome (Randich and Brun, 2015). Thus, unicellular cyanobacterial strains adopted different strategies for PG synthesis during cell growth.

### The Filamentous Cyanobacteria *Arthrospira* and *Oscillatoria* Depend on Both Septal and Peripheral PG Synthesis for Cell Growth

**FIGURE 2:**
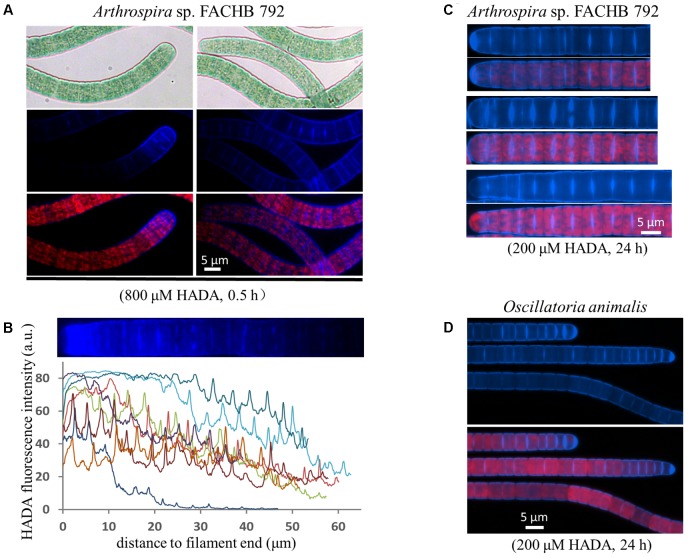
Growth pattern of the filamentous cyanobacteria *Arthrospira* sp. FACHB 792 and *Oscillatoria animalis*. **(A,C)** *Arthrospira* pulse-labeled with 800 μM of HADA for half an hour or long-term labeled with 200 μM of HADA for 24 h. **(B)** The HADA signal at the tips of some pulse labeled *Arthrospira* quantified with ImageJ. Every curve in the graph represents the fluorescence intensity along one end of a filament as exemplified above the graph. The filaments of long-term labeled *Arthrospira* were straightened with ImageJ. **(D)** *Oscillatoria* incubated with 200 μM of HADA for 24 h. Bright-field photos (gray background pictures in panel A), as well as images of HADA fluorescence (blue), photosynthetic pigment fluorescence (red), and their merges are shown.

We used two filamentous cyanobacteria, which do not fix nitrogen, as examples to examine the cell growth pattern by HADA labeling. The cells of both strains have a disc shape growing much shorter than wide (**Figure 2**). For *Arthrospira*, we first performed a short-time pulse labeling of 30 min with 800 μM of HADA. At the tip of many filaments, a high labeling zone, covering several cells could be observed (**Figure 2A**), and quantification of the HADA signal revealed stronger PG synthesis at the end of these filaments (**Figure 2B**). In general, both septal and peripheral PG synthesis could be seen, indicating that the cell growth requires both lateral and septal PG synthesis during cell growth, although much stronger HADA labeling occurred at the newly formed division site just separating two daughter cells (**Figure 2C**). *Oscillatoria* displayed a similar PG synthesis pattern as *Arthrospira* (**Figure 2D**), with both a lateral and septal HADA incorporation.

**FIGURE 3:**
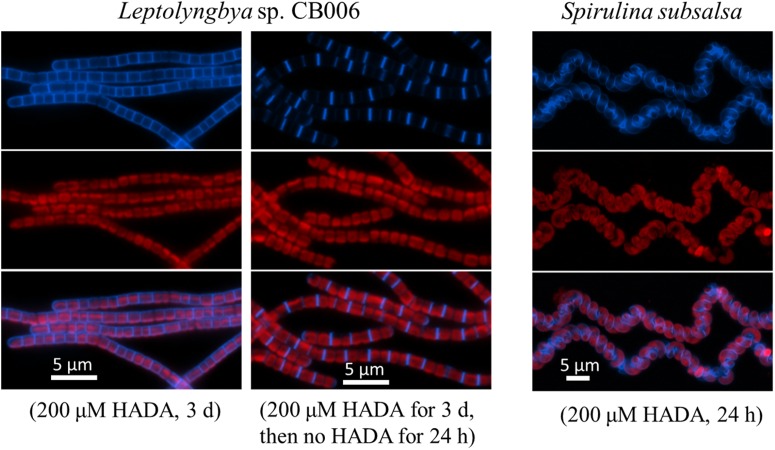
HADA incorporation into *Leptolyngbya* sp. CB006 and *Spirulina subsalsa. Leptolyngbya* requires a longer incubation time with HADA. Photos of HADA fluorescence (blue), photosynthetic pigment fluorescence (red), and their merges are shown.

Similar pattern of PG synthesis could also be observed with a *Leptolyngbya* species (with rod-shaped cells along the filaments) and a *Spirulina* species (twisted cells along the filaments); although the peripheral HADA signal in these species appeared weaker than in *Oscillatoria* and *Arthrospira* (**Figure 3**). HADA pattern in *Spirulina* was twisted along the filament, mirroring the shape of the filaments. The *Leptolyngbya* species grew with a doubling time of approximately 13 h but required a much longer incubation time to visualize HADA incorporation (3 days). At 24 h after transferring cells into HADA-free medium, HADA fluorescence at the septal sites mostly still remained, whereas those at the peripheral disappeared, indicating that PG turnover was faster at the cell periphery than at the cell division site in this strain.

### Cell Growth and Division in the N_2_-Fixing Filamentous Strain *Anabaena*

**FIGURE 4:**
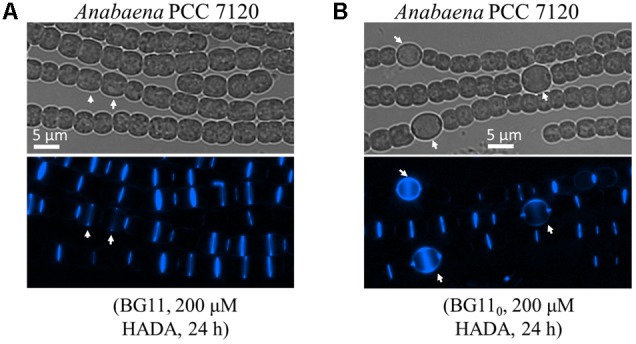
PG synthesis during vegetative growth and heterocyst development in *Anabaena* PCC 7120. Filaments of *Anabaena* were grown in BG11 using nitrate as a combined nitrogen source, then incubated with 200 μM of HADA in BG11 **(A)**, or in BG11_0_ **(B)** to induce heterocyst development. Arrows in A indicate elongated cells that just started to incorporate HADA at the division site, and in B heterocysts. Bright-field (gray) and HADA fluorescence (blue) images are shown.

*Anabaena* PCC 7120 is a model strain for the studies of heterocyst development. We thus used this organism to trace the pattern of PG synthesis during both vegetative growth and heterocyst development. We first incubated filaments of *Anabaena* with various concentrations of HADA for different times and found that labeling time contributed to labeling efficiency much more than HADA concentration. For instance, 3 h of pulse labeling only gave a dim HADA signal at a few cell junctions irrespective of the used concentration of HADA, whereas 9 h of incubation with 200 μM HADA could gave bright labeling (data not shown). Therefore, we used a period of 9–24 h, for efficient labeling in *Anabaena* in subsequent experiments with 200 μM of HADA. Under vegetative growth, an intensive HADA signal could be detected in young cells at the division site, or old cells already finished cell constriction; at the same time, a weak but detectable HADA signal at the cell periphery could also be seen (**Figure 4A**).

**FIGURE 5:**
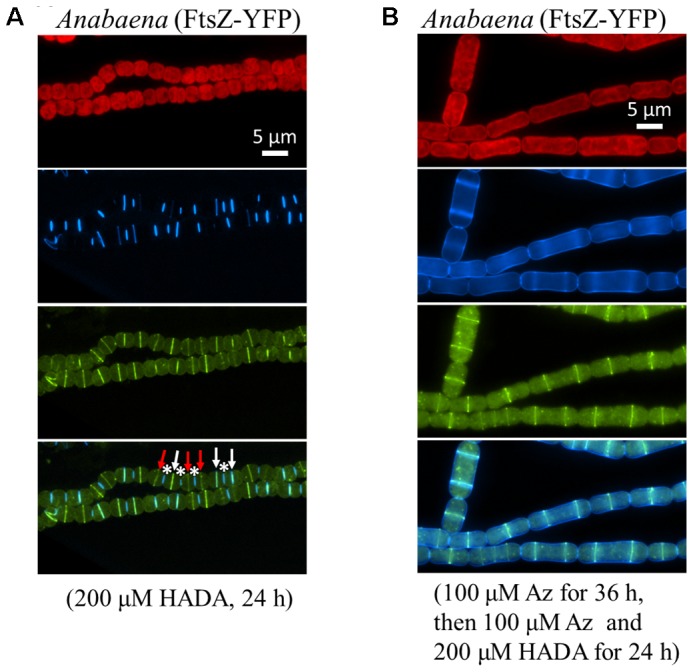
Relationship between cell division and PG synthesis in *Anabaena* PCC 7120. **(A)** An *Anabaena* strain in which the cell division gene *ftsZ* was replaced by a *ftsZ-yfp* translational fusion was treated with HADA, and imaged under a fluorescence microscope. The top panel shows red photosynthetic pigment and the HADA signal in blue; the middle panel shows the Z-ring in yellow; and the bottom panel shows a superimposition of the same filaments with both HADA and FtsZ-YFP signals. Asterisks indicate cell–cell junctions labeled by HADA, white arrows indicate septa having both HADA signal and FtsZ-YFP signal, and red arrows indicate septa having FtsZ-YFP signal only. **(B)** The same recombinant strain as in A was treated with aztreonam (Az), an antibiotic targeting to FtsI, a PG synthase involved in cell division. Cells elongated as a consequence of cell division inhibition as previously reported (Sakr et al., 2006). The same filaments were pictured in red (photosynthetic pigments), blue (HADA), yellow (FtsZ-YFP) and superimposition of the HADA and YFP signals.

FtsZ, as the cell division initiating protein, assembles into Z-ring at the mid cell during the early stage of cell division. To explore the relationship between cell division and PG synthesis, we first probed the PG synthesis with HADA labeling in a strain in which FtsZ is fused to YFP (**Figure 5**). Three types of labeling could be observed. First, there were cells containing a Z-ring at the mid-cell position, but no detectable incorporation of HADA (red arrows, **Figure 5A**) or sign of cell constriction were observed. Second, both FtsZ-YFP and HADA fluorescence could be observed at the mid-cell position, with the two fluorescence signals superimposed (white arrows). Some of these cells did not show evidence of cell constriction, whereas others already evinced signs of cell constriction, indicating that cell division progressed to the constriction stage. Finally, some cells in which cell division was completed, with a narrow cell constriction between two cells, gave only a HADA signal but no FtsZ-YFP fluorescence (asterisks). At the end of the cell division, the Z-ring disassembled at the division site, but HADA incorporated at the constriction site remained for a long time, indicating a slow turnover of PG at the septa. Consistent with these observations, even after 72 h of HADA removal, HADA fluorescence could still be observed at many cell–cell junctions (data not shown). The antibiotic aztreonam targets the cell division protein FtsI/PBP3 which is involved in PG synthesis at the bacterial division site. In the presence of aztreonam, cell division could be impaired, leading to elongated forms of cells. Under such conditions, FtsZ-YFP could still be observed as a ring structure, but a HADA incorporation at the peripheral sidewall became obvious (**Figure 5B**).

### Regulation of PG Synthesis During Heterocyst Development

**FIGURE 6:**
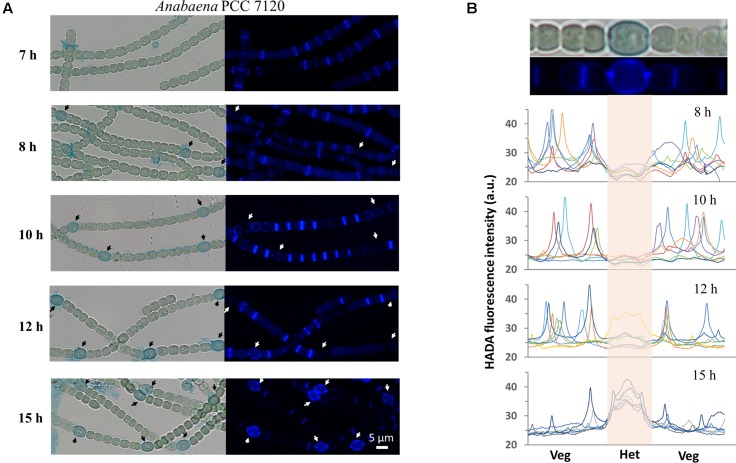
Determination of the timing of PG synthesis in heterocysts. Hetercoysts differentiation takes approximately 20–24 h after the deprivation of combined nitrogen from the growth medium. Heterocysts were induced in the BG11_0_ medium containing 200 μM HADA. Seven hours after the induction, filaments were stained with Alcian blue which binds to heterocyst-specific polysaccharide layer, to better recognize the developing cells (arrows). **(A)** The filaments were examined under both bright-field and for HADA fluorescence to determine the incorporation of HADA into developing heterocysts. The same procedure was followed after 10 h, 12 h and 15 h of induction. All HADA fluorescence images were taken using the same exposure time (1 s) and were processed with the same procedures using ImageJ. **(B)** Quantification of HADA incorporation in developing and mature heterocysts at 8 h, 10 h, 12 h, and 15 h. At each time point, the fluorescence signal along seven representative short filaments, each of which consists of one heterocyst (Het) at the center and a few vegetative cells (Veg) on each side, were quantified with ImageJ. Every curve in the graphs represents the fluorescence intensity along one filament. The signal from developing or mature heterocysts in the graphs is indicated by the center shadow area. The bright-field microscopy image and the HADA fluorescence picture of one filament from the 15 h sample were shown at the top of the panel for illustration purpose.

As shown above, PG synthesis in vegetative cells was mainly at the division or constriction site. However, as terminally differentiated cells developed from vegetative cells, heterocysts are incapable of cell division. To explore whether PG synthesis is still active in heterocysts, we induced heterocysts by growing vegetative filaments of *Anabaena* in a nitrogen-free medium containing 200 μM HADA, and measured HADA fluorescence in mature heterocysts after 24 h of induction. As shown in **Figure 4B**, intensive HADA incorporation in heterocysts was observed at the central peripheral areas, as well as at the polar regions, with PG labeling protruding to neighboring vegetative cells. Overall, HADA fluorescence intensity was much stronger in mature heterocysts than in vegetative cells. Because PG synthesis appeared to be upregulated in heterocysts, we determined the timing of PG synthesis during heterocyst development (**Figure 6**). Alcian blue is a dye that can specifically stain the heterocyst polysaccharide layer, even at the early phase of heterocyst development before any morphological signs. As shown in **Figure 6A**, whereas no Alcian blue staining could be observed in filaments 7 h after the initiation of heterocyst development, some cells were already weakly stained at 8 h, indicating that the polysaccharide layer began to be deposited at the cell wall. From 10 h on, clear Alcian blue staining could be observed in the developing cells, indicative of proheterocyst formation. At 12 h, very few proheterocysts gave rise to HADA fluorescence, suggesting that some had begun to synthesize PG at the cell wall. At 15 h, all proheterocysts showed increased HADA incorporation, indicating that active PG synthesis occurred in these cells. This observation was consistent with the quantification of the HADA signal in a representative number of differentiating cells at these time points (**Figure 6B**).

## Discussion

In this study, we investigated the PG synthesis patterns by HADA staining in several cyanobacterial strains which cover most of the common morphological types known in cyanobacteria: unicellular, filamentous, spiral, or heterocystous. We also tested several strains that grow in colonial or branching-filamentous forms, but the existence of a gelatinous sheath surrounding the cells prevented a proper staining of PG by HADA incorporation (data not shown). Most of the commonly found cell morphologies in cyanobacteria are represented in this study. Despite the diversity of cell size and morphology, the PG synthesis patterns observed in cyanobacteria share some common features and may display a distinct characteristic depending on the strain examined. First, as a general rule, all the cyanobacterial strains we examined thus far showed intensive HADA incorporation at the septal site. Once incorporated at the division site, HADA remained for a long time after transferring cells into HADA-free medium, which is evidence of a slow turnover. Second, peripheral PG synthesis (or lateral elongation) was also common in cyanobacteria, but varied extensively: some strains such as *Leptolyngbya* required a long incubation with HADA to reveal the lateral PG synthesis, whereas other strains such as *Synechocystis* or *Anabaena* gave an HADA signal distributed around the cell at a similar intensity (see *Arthrospira*, *Leptolyngbya*, and *Oscillatoria*, for example). HADA signal intensity may correlate with the degree of septation after cell division, (i.e., the degree of polar morphological remodeling). Indeed, when the two daughter cells were either completely separated after cell division (unicellular strains such as *Synechocystis* for example), or cell constriction was severe with a much narrower connection at cell–cell junctions, the cell poles generated during cell division required extensive PG hydrolysis and resynthesis in order to be remodeled. In contrast, in those strains whose cell constriction was limited, PG synthesis was restricted mainly to the separation of the two daughter cells. Beyond these general rules of PG synthesis, some cyanobacterial strains displayed distinct features: *Microcystis* had a preseptal-type growth mode, similar as that observed in *Caulobacter crescentus* (Aaron et al., 2007); *Arthrospira* and *Oscillatoria* filaments often displayed a stronger, or even graded HADA signal intensity at the tip of the filaments. Thus, cyanobacteria show great diversity in term of cell growth mode.

Using *Anabaena*, we examined PG synthesis in vegetative cells and its relationship to cell division. The data suggested that HADA incorporation and consequently, PG synthesis, occurred at the division site from the onset until polar morphogenesis, as well as weakly along the long axis of the cell wall (**Figure 4A**). In some larger vegetative cells, corresponding to those ready to divide, a faint line of HADA fluorescence at the mid-cell position could be seen before cell constriction (white arrows in **Figure 4A**), suggesting that the PG synthesis at the division site starts relatively early during the cell cycle. Furthermore, synthesis of PG at the division site likely followed the direction of FtsZ. This was suggested by the evidence that only a Z-ring, but no HADA signal, was found at the septum in many cells without constriction formation, whereas a Z-ring superimposed with HADA signal appeared in all cells that had begun constricting (**Figure 5A**). The superimposition of the Z-ring and HADA incorporation activity at cell septum implies cell division and HADA incorporation are highly coordinated. When cell division was inhibited by aztreonam treatment, cells became large and elongated. In most of these cells, HADA incorporation was no longer restricted to the Z-ring site. Rather, it occurred on the entire cell surface (**Figure 5B**). Thus, PG synthesis and cell division can be uncoupled at the septum when cell division is blocked.

Heterocysts are differentiated from vegetative cells upon combined nitrogen deprivation. Compared to vegetative cells, heterocysts are larger and feature a thick cell envelope with two additional layers: an inner glycolipid layer and an outer polysaccharide layer (Wolk, 1996). We found that PG synthesis in differentiating cells (proheterocysts) became active after the formation of the polysaccharide layer, at a relatively late stage of heterocyst development (**Figure 6**). HADA labeling occurred around the whole proheterocyst cells, in contrast to vegetative cells, where labeling was mainly noted at the division site. The HADA signal in proheterocysts covered a large central area, which corresponds to a typical lateral growth mode found in *E. coli* or *B. subtilis*. This is also similar to the elongation mode of vegetative cells when septal PG synthesis is inhibited by aztreonam. The cell division protein FtsZ is known to be downregulated during heterocyst development at both transcriptional and post-transcriptional levels (Kuhn et al., 2000; Wang and Xu, 2005). Thus, the PG synthesis pattern in proheterocysts is likely to be independent of FtsZ but dependent on MreB, which has been reported to be upregulated in proheterocysts (Hu et al., 2007). The regulation of PG synthesis is consistent with the finding that several PG synthesis enzymes are required for heterocyst development or functioning (Lázaro et al., 2001; Zhu et al., 2001; Lehner et al., 2011; Berendt et al., 2012; Videau et al., 2016; Bornikoel et al., 2017; Zheng et al., 2017). Based on our findings and previous reports, we propose that a thick PG layer is another characteristic of mature heterocysts, in addition to their glycolipid and polysaccharide layers. Additionally, some of the enzymes involved in PG metabolism may affect the functioning of heterocysts by modulating PG synthesis during heterocyst maturation, in addition to their roles in cell–cell communication.

The fluorescent derivative of vancomycin, Van-FL, was previously used to stain PG in the heterocyst-forming cyanobacteria *Anabaena* PCC 7120 and *Nostoc punctiforme* (Lehner et al., 2013; Rudolf et al., 2015; Mariscal et al., 2016). The staining of vegetative cells with Van-FL showed similar patterns to that of HADA, with intensive septal labeling. However, the Van-FL signal did not increase in heterocysts, which differs from our observations using HADA in this study. Two reasons may contribute to this difference. First, Van-FL is an analog of the antibiotic vancomycin. Thus, it is unsuitable to follow the entire process of heterocyst development, which lasts for more than 20 h, with live-cell imaging. Second, HADA incorporation occurs at the late stage of heterocyst development and Van-FL is a relatively large molecule (1921 Da) compared to HADA (292 Da). Thus, it is more difficult for Van-FL to penetrate the already formed thick proheterocyst envelope and outer membrane to reach PG synthesis sites during the staining procedure. In conclusion, HADA is a more suitable labeling tool for the study of heterocyst development.

## Author Contributions

C-CZ, J-YZ, and G-ML designed the study and analyzed the data. J-YZ, G-ML, and W-YX performed the experiments. C-CZ and J-YZ wrote the manuscript.

## Conflict of Interest Statement

The authors declare that the research was conducted in the absence of any commercial or financial relationships that could be construed as a potential conflict of interest.

## References

[B1] Aaron M. Charbon G. Lam H. Schwarz H. Vollmer W. Jacobs-Wagner C. (2007). The tubulin homologue FtsZ contributes to cell elongation by guiding cell wall precursor synthesis in *Caulobacter crescentus*. *Mol. Microbiol.* 64 938–952. 10.1111/j.1365-2958.2007.05720.x 17501919

[B2] Aiba S. Ogawa T. (1977). Assessment of growth yield of a blue — green alga, *Spirulina platensis*, in axenic and continuous culture. *Microbiology* 102 179–182. 10.1099/00221287-102-1-179

[B3] Bekker A. Holland H. D. Wang P. L. Rumble D. Stein H. J. Hannah J. L. (2004). Dating the rise of atmospheric oxygen. *Nature* 427 117–120. 10.1038/nature02260 14712267

[B4] Berendt S. Lehner J. Zhang Y. V. Rasse T. M. Forchhammer K. Maldener I. (2012). Cell wall amidase AmiC1 is required for cellular communication and heterocyst development in the cyanobacterium *Anabaena* PCC 7120 but not for filament integrity. *J. Bacteriol.* 194 5218–5227. 10.1128/JB.00912-12 22821973 PMC3457231

[B5] Bisson-Filho A. W. Hsu Y. P. Squyres G. R. Kuru E. Wu F. Jukes C. (2017). Treadmilling by FtsZ filaments drives peptidoglycan synthesis and bacterial cell division. *Science* 355 739–743. 10.1126/science.aak9973 28209898 PMC5485650

[B6] Bornikoel J. Carrión A. Fan Q. Flores E. Forchhammer K. Mariscal V. (2017). Role of two cell wall amidases in septal junction and nanopore formation in the multicellular cyanobacterium *Anabaena* sp. PCC 7120. *Front. Cell. Infect. Microbiol.* 7:386. 10.3389/fcimb.2017.00386 28929086 PMC5591844

[B7] Busiek K. K. Margolin W. (2015). Bacterial actin and tubulin homologs in cell growth and division. *Curr. Biol.* 25 R243–R254. 10.1016/j.cub.2015.01.030 25784047 PMC5519336

[B8] Cai Y. P. Wolk C. P. (1990). Use of a conditionally lethal gene in *Anabaena* sp. strain PCC 7120 to select for double recombinants and to entrap insertion sequences. *J. Bacteriol.* 172 3138–3145. 10.1128/jb.172.6.3138-3145.1990 2160938 PMC209118

[B9] Dittmann E. Wiegand C. (2006). Cyanobacterial toxins–occurrence, biosynthesis and impact on human affairs. *Mol. Nutr. Food Res.* 50 7–17. 10.1002/mnfr.200500162 16304634

[B10] Divakaruni A. V. Baida C. White C. L. Gober J. W. (2007). The cell shape proteins MreB and MreC control cell morphogenesis by positioning cell wall synthetic complexes. *Mol. Microbiol.* 66 174–188. 10.1111/j.1365-2958.2007.05910.x 17880425

[B11] Elhai J. Vepritskiy A. Muro-Pastor A. M. Flores E. Wolk C. P. (1997). Reduction of conjugal transfer efficiency by three restriction activities of *Anabaena* sp. strain PCC 7120. *J. Bacteriol.* 179 1998–2005. 9068647 10.1128/jb.179.6.1998-2005.1997 PMC178925

[B12] Herrero A. Stavans J. Flores E. (2016). The multicellular nature of filamentous heterocyst-forming cyanobacteria. *FEMS Microbiol. Rev.* 40 831–854. 10.1093/femsre/fuw029 28204529

[B13] Hoiczyk E. Hansel A. (2000). Cyanobacterial cell walls: news from an unusual prokaryotic envelope. *J. Bacteriol.* 182 1191–1199. 10.1128/JB.182.5.1191-1199.2000 10671437 PMC94402

[B14] Hu B. Yang G. Zhao W. Zhang Y. Zhao J. (2007). MreB is important for cell shape but not for chromosome segregation of the filamentous cyanobacterium *Anabaena* sp. PCC 7120. *Mol. Microbiol.* 63 1640–1652. 10.1111/j.1365-2958.2007.05618.x 17367385

[B15] Jeltsch A. (2013). Oxygen, epigenetic signaling, and the evolution of early life. *Trends Biochem. Sci.* 38 172–176. 10.1016/j.tibs.2013.02.001 23454078

[B16] Kuhn I. Peng L. Bedu S. Zhang C. C. (2000). Developmental regulation of the cell division protein FtsZ in *Anabaena* sp. strain PCC 7120, a cyanobacterium capable of terminal differentiation. *J. Bacteriol.* 182 4640–4643. 10.1128/JB.182.16.4640-4643.2000 10913101 PMC94639

[B17] Kuru E. Tekkam S. Hall E. Brun Y. V. Van Nieuwenhze M. S. (2015). Synthesis of fluorescent D-amino acids and their use for probing peptidoglycan synthesis and bacterial growth in situ. *Nat. Protoc.* 10 33–52. 10.1038/nprot.2014.197 25474031 PMC4300143

[B18] Lázaro S. Fernández-Piñas F. Fernández-Valiente E. Blanco-Rivero A. Leganés F. (2001). *pbpB*, a gene coding for a putative penicillin-binding protein, is required for aerobic nitrogen fixation in the cyanobacterium *Anabaena* sp. strain PCC7120. *J. Bacteriol.* 183 628–636. 10.1128/JB.183.2.628-636.2001 11133957 PMC94919

[B19] Lehner J. Berendt S. Dörsam B. Pérez R. Forchhammer K. Maldener I. (2013). Prokaryotic multicellularity: a nanopore array for bacterial cell communication. *FASEB J.* 27 2293–2300. 10.1096/fj.12-225854 23444428

[B20] Lehner J. Zhang Y. Berendt S. Rasse T. M. Forchhammer K. Maldener I. (2011). The morphogene AmiC2 is pivotal for multicellular development in the cyanobacterium *Nostoc punctiforme*. *Mol. Microbiol.* 79 1655–1669. 10.1111/j.1365-2958.2011.07554.x 21244533

[B21] Marbouty M. Saguez C. Cassier-Chauvat C. Chauvat F. (2009). Characterization of the FtsZ-interacting septal proteins SepF and Ftn6 in the spherical-celled cyanobacterium *Synechocystis* strain PCC 6803. *J. Bacteriol.* 191 6178–6185. 10.1128/JB.00723-09 19648234 PMC2747883

[B22] Mariscal V. Nürnberg D. J. Herrero A. Mullineaux C. W. Flores E. (2016). Overexpression of SepJ alters septal morphology and heterocyst pattern regulated by diffusible signals in *Anabaena*. *Mol. Microbiol.* 101 968–981. 10.1111/mmi.13436 27273832

[B23] Nicolaisen K. Hahn A. Schleiff E. (2009). The cell wall in heterocyst formation by *Anabaena* sp. PCC 7120. *J. Basic Microbiol.* 49 5–24. 10.1002/jobm.200800300 19253332

[B24] Ponce-Toledo R. I. Deschamps P. López-García P. Zivanovic Y. Benzerara K. Moreira D. (2017). An early-branching freshwater cyanobacterium at the origin of plastids. *Curr. Biol.* 27 386–391. 10.1016/j.cub.2016.11.056 28132810 PMC5650054

[B25] Prentki P. Krisch H. M. (1984). In vitro insertional mutagenesis with a selectable DNA fragment. *Gene* 29 303–313. 10.1016/0378-1119(84)90059-3 6237955

[B26] Randich A. M. Brun Y. V. (2015). Molecular mechanisms for the evolution of bacterial morphologies and growth modes. *Front. Microbiol.* 6:580. 10.3389/fmicb.2015.00580 26106381 PMC4460556

[B27] Rudolf M. Tetik N. Ramos-León F. Flinner N. Ngo G. Stevanovic M. (2015). The peptidoglycan-binding protein SjcF1 influences septal junction function and channel formation in the filamentous cyanobacterium *Anabaena*. *mBio* 6:e00376–15. 10.1128/mBio.00376-15 26126850 PMC4488944

[B28] Sakr S. Jeanjean R. Zhang C. C. Arcondeguy T. (2006). Inhibition of cell division suppresses heterocyst development in *Anabaena* sp. strain PCC 7120. *J. Bacteriol.* 188 1396–1404. 10.1128/JB.188.4.1396-1404.2006 16452422 PMC1367218

[B29] Schneider C. A. Rasband W. S. Eliceiri K. W. (2012). NIH Image to ImageJ: 25 years of image analysis. *Nat. Methods* 9 671–675. 10.1038/nmeth.2089 22930834 PMC5554542

[B30] Stanier R. Y. Kunisawa R. Mandel M. Cohen-Bazire G. (1971). Purification and properties of unicellular blue-green algae (order Chroococcales). *Bacteriol. Rev.* 35 171–205. 4998365 10.1128/br.35.2.171-205.1971 PMC378380

[B31] Tiyanont K. Doan T. Lazarus M. B. Fang X. Rudner D. Z. Walker S. (2006). Imaging peptidoglycan biosynthesis in *Bacillus subtilis* with fluorescent antibiotics. *Proc. Natl. Acad. Sci. U.S.A.* 103 11033–11038. 10.1073/pnas.0600829103 16832063 PMC1544169

[B32] Videau P. Rivers O. S. Ushijima B. Oshiro R. T. Kim M. J. Philmus B. (2016). Mutation of the *murC* and *murB* genes impairs heterocyst differentiation in *Anabaena* sp. strain PCC 7120. *J. Bacteriol.* 198 1196–1206. 10.1128/JB.01027-15 26811320 PMC4859589

[B33] Wang Y. Xu X. (2005). Regulation by *hetC* of genes required for heterocyst differentiation and cell division in *Anabaena* sp. strain PCC 7120. *J. Bacteriol.* 187 8489–8493. 10.1128/JB.187.24.8489-8493.2005 16321953 PMC1316993

[B34] Wolk C. P. (1996). Heterocyst formation. *Annu. Rev. Genet.* 30 59–78. 10.1146/annurev.genet.30.1.59 8982449

[B35] Yang X. Lyu Z. Miguel A. McQuillen R. Huang K. C. Xiao J. (2017). GTPase activity-coupled treadmilling of the bacterial tubulin FtsZ organizes septal cell wall synthesis. *Science* 355 744–747. 10.1126/science.aak9995 28209899 PMC5851775

[B36] Zhang C. C. Huguenin S. Friry A. (1995). Analysis of genes encoding the cell division protein FtsZ and a glutathione synthetase homologue in the cyanobacterium *Anabaena* sp. PCC 7120. *Res. Microbiol.* 146 445–455. 8525061 10.1016/0923-2508(96)80290-7

[B37] Zhang C. C. Laurent S. Sakr S. Peng L. Bédu S. (2006). Heterocyst differentiation and pattern formation in cyanobacteria: a chorus of signals. *Mol. Microbiol.* 59 367–375. 10.1111/j.1365-2958.2005.04979.x 16390435

[B38] Zheng Z. Omairi-Nasser A. Li X. Dong C. Lin Y. Haselkorn R. (2017). An amidase is required for proper intercellular communication in the filamentous cyanobacterium *Anabaena* sp. PCC 7120. *Proc. Natl. Acad. Sci. U.S.A.* 114 E1405–E1412. 10.1073/pnas.1621424114 28159891 PMC5338405

[B39] Zhu J. Jäger K. Black T. Zarka K. Koksharova O. Wolk C. P. (2001). HcwA, an autolysin, is required for heterocyst maturation in *Anabaena* sp. strain PCC 7120. *J. Bacteriol.* 183 6841–6851. 10.1128/JB.183.23.6841-6851.2001 11698373 PMC95525

