# Supplementary material for: Diversity of Growth Patterns Probed in Live Cyanobacterial Cells Using a Fluorescent Analog of a Peptidoglycan Precursor
Source: Front Microbiol. 2018 Apr 24;9:791. doi: 10.3389/fmicb.2018.00791 (PMC5928242; doi:10.3389/fmicb.2018.00791)
Supplement: Supplementary file 1 [file Data_Sheet_1.PDF]

Supplementary Material

Diversity of growth patterns probed in live cyanobacterial cells using a fluorescent analog of a peptidoglycan precursor

Ju-Yuan Zhang, Gui-Ming Lin, Wei-Yue Xing, Cheng-Cai Zhang*

*** Correspondence:** Corresponding Author: cczhang@ihb.ac.cn

Supplementary Table 1. The primers used in this study.

Name	Nucleotide Sequence (5' to 3')	Tm (°C)	Purpose
PCINT2a	tcgatgagtcgactctagctagaggatcg	67.61	To amplify pRL271. The 5450 bp product was circularized to create pCint2.
PCINT2b	gagtcgactcatcgaataaatacctgtgac	64.88	
PspF214m	atgcaccttgccgtagaaggctcacgcaactggtccagaac	78.92	To amplify the Sp/Sm resistance gene from the omega fragment. The 1053 bp product was used to make pSYFP2-sp.
PspR797	cattcaaaaggtcatccacgggagacattatttgccgactacct	74.64	
Psyfp2spF	ggatccaaggcctctggtggatctg	67.99	To amplify *syfp2* ORF and Sp/Sm resistance gene from pSYFP2-sp. The 2124 bp product was used to make pFtsZ-SYFP2.
Psyfp2spR	gggagtactgatgatccggtgatt	63.30	
Palr3858F160	gcagaaattcgatatctagatcgtattggcgagattgttcctgg	72.41	To amplify an *ftsZ* region (160th to 1284th) from *Anabaena* chromosome. The 1167 bp product was used to make pFtsZ-SYFP2.
Palr3858R1284	tccaccagaggccttggatccatttttgggtggtcgccgtc	79.95	
Palr3858F1353	accggatcatcagtactccctgctaattttcaagttcagaggt	73.87	To amplify an *ftsZ* region (1353rd to 2377th) from *Anabaena* chromosome. The 1062 bp product was used to make pFtsZ-SYFP2.
Palr3858R2377	cgcaacgttgttgccattgcaggtagaacttgtaccagtgca	77.15	

Supplementary Figures

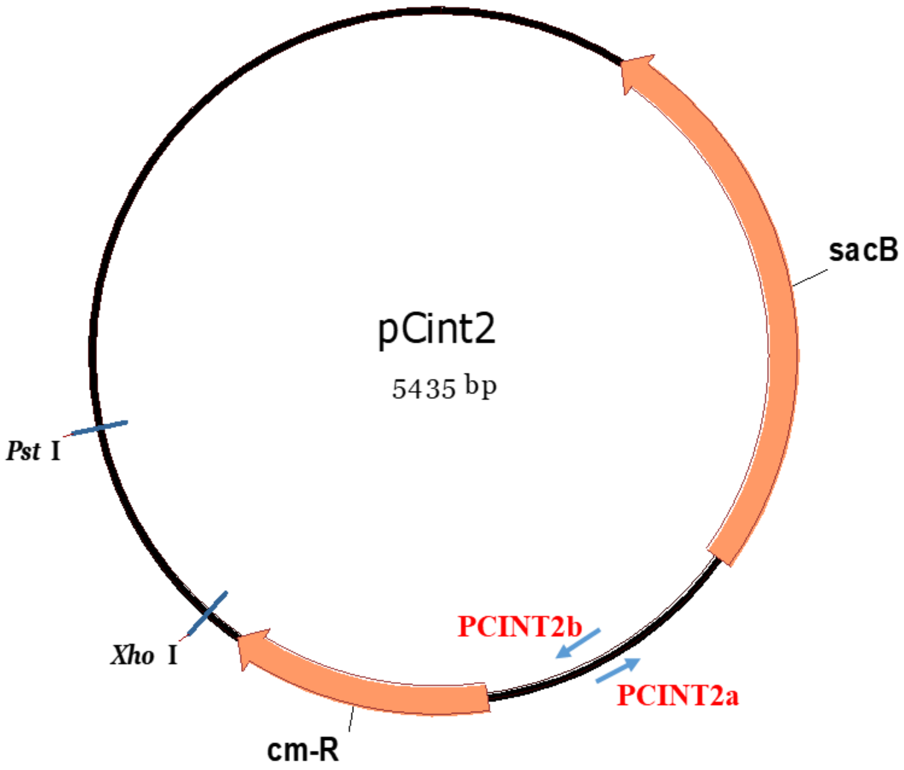

Supplementary Figure 1. The map of the plasmid pCint2. The sequence of pCint2 has been deposited into GenBank (accession number: MH050934). The matching sites of the primers PCINT2a and PCINT2b are indicated as arrows. cm-R, chloramphenicol resistance gene; sacB, the gene encoding *Bacillus subtilis* levansucrase.

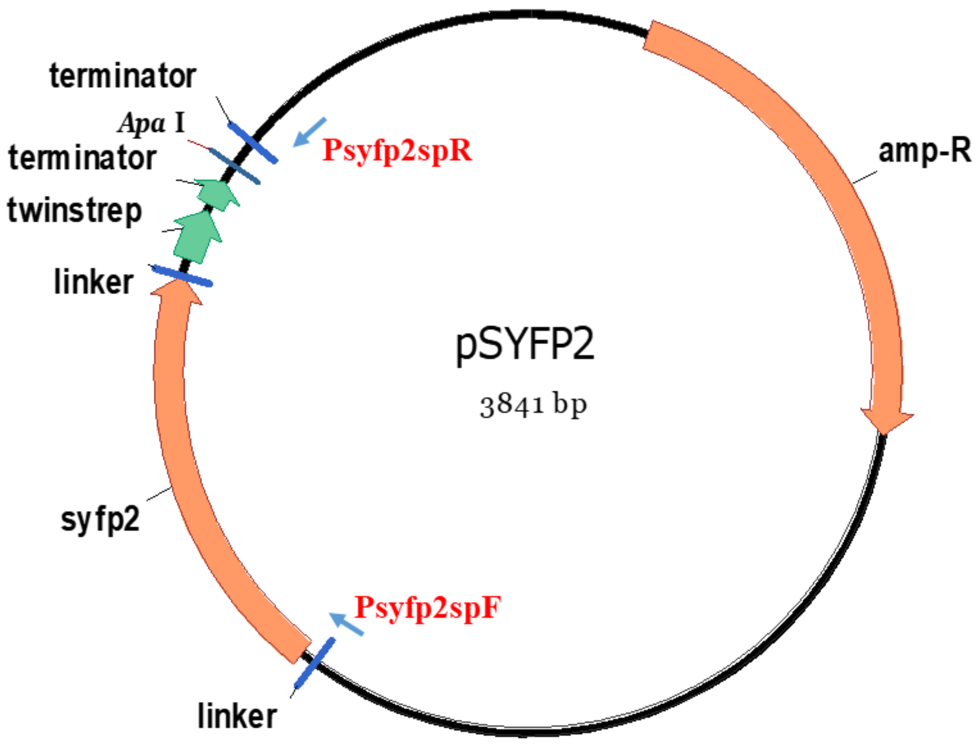

Supplementary Figure 2. The map of the plasmid pSYFP2. The sequence of pSYFP2 has been deposited into GenBank (accession number: MH050935). The matching sites of the primers Psyfp2spF and Psyfp2spR are indicated as arrows. amp-R, ampicillin resistance gene; syfp2, the ORF encoding the YFP variant SYFP2.

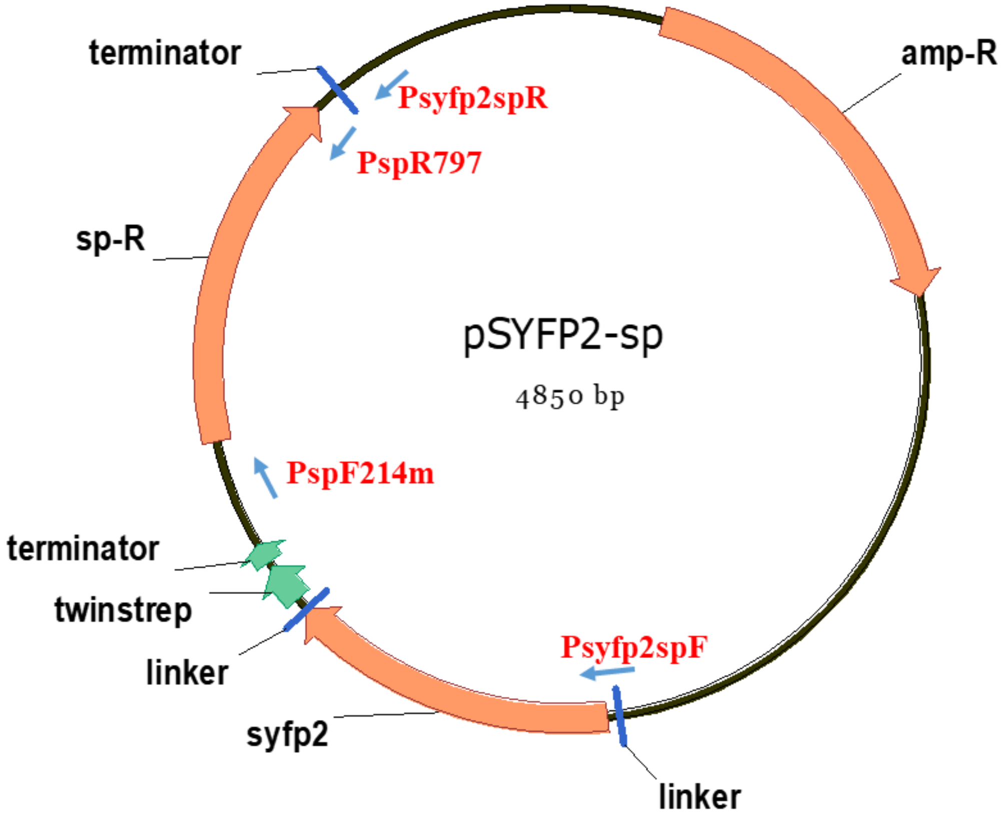

Supplementary Figure 3. The map of the plasmid pSYFP2-sp. The sequence of pSYFP2-sp has been deposited into GenBank (accession number: MH050936). The matching sites of the primers PspF214m, Psp797R, Psyfp2spF and Psyfp2spR are indicated as arrows. sp-R, spectinomycin/streptomycin resistance gene; amp-R, ampicillin resistance gene; syfp2, the ORF encoding the YFP variant SYFP2.

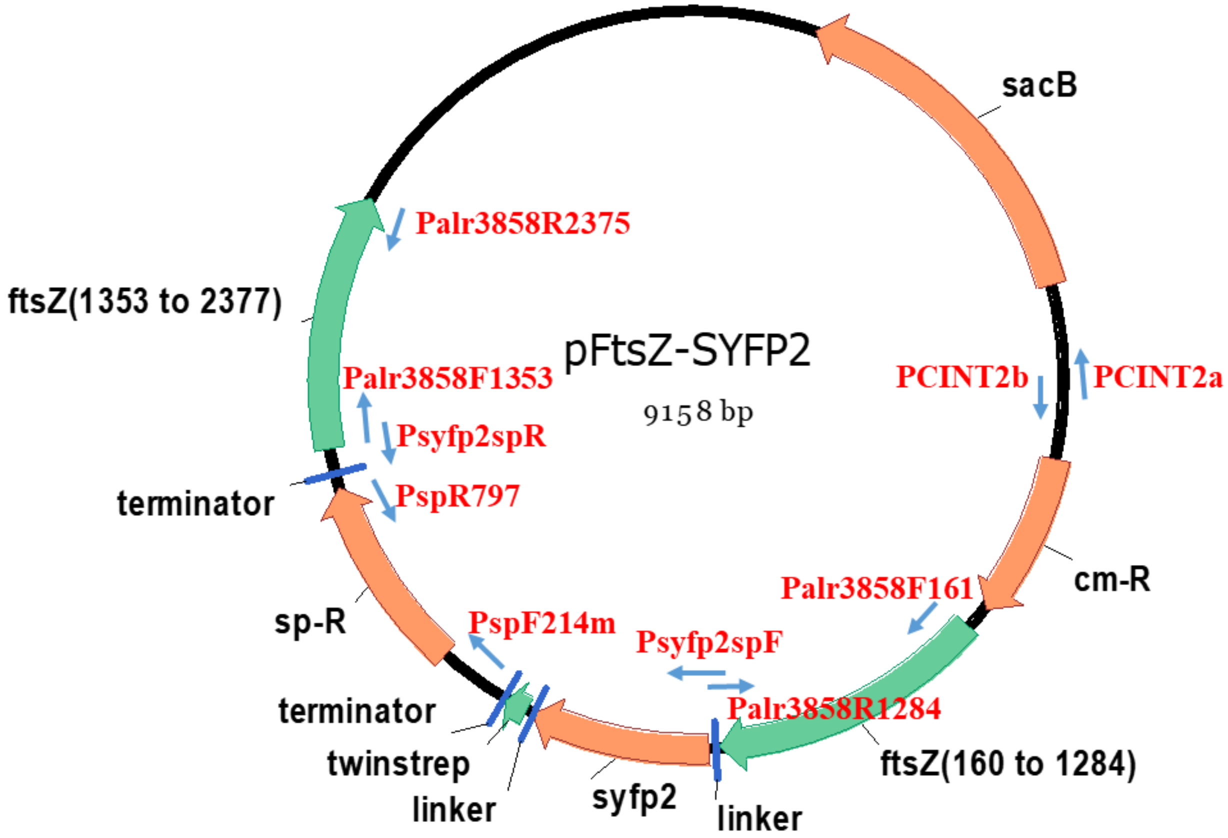

Supplementary Figure 4. The map of the plasmid pFtsZ-SYFP2. The sequence of pFtsZ-SYFP2 has been deposited into GenBank (accession number: MH050937). The matching sites of the primers PCINT2a, PCINT2b, PspF214m, Psp797R, Psyfp2spF, Psyfp2spR, Palr3858F161, Palr3858R1284, Palr3858F1353 and Palr3858R2375 are indicated as arrows. sp-R, spectinomycin/streptomycin resistance gene; amp-R, ampicillin resistance gene; syfp2, the ORF encoding the YFP variant SYFP2; cm-R, chloramphenicol resistance gene; sacB, the gene encoding *Bacillus subtilis* levansucrase.

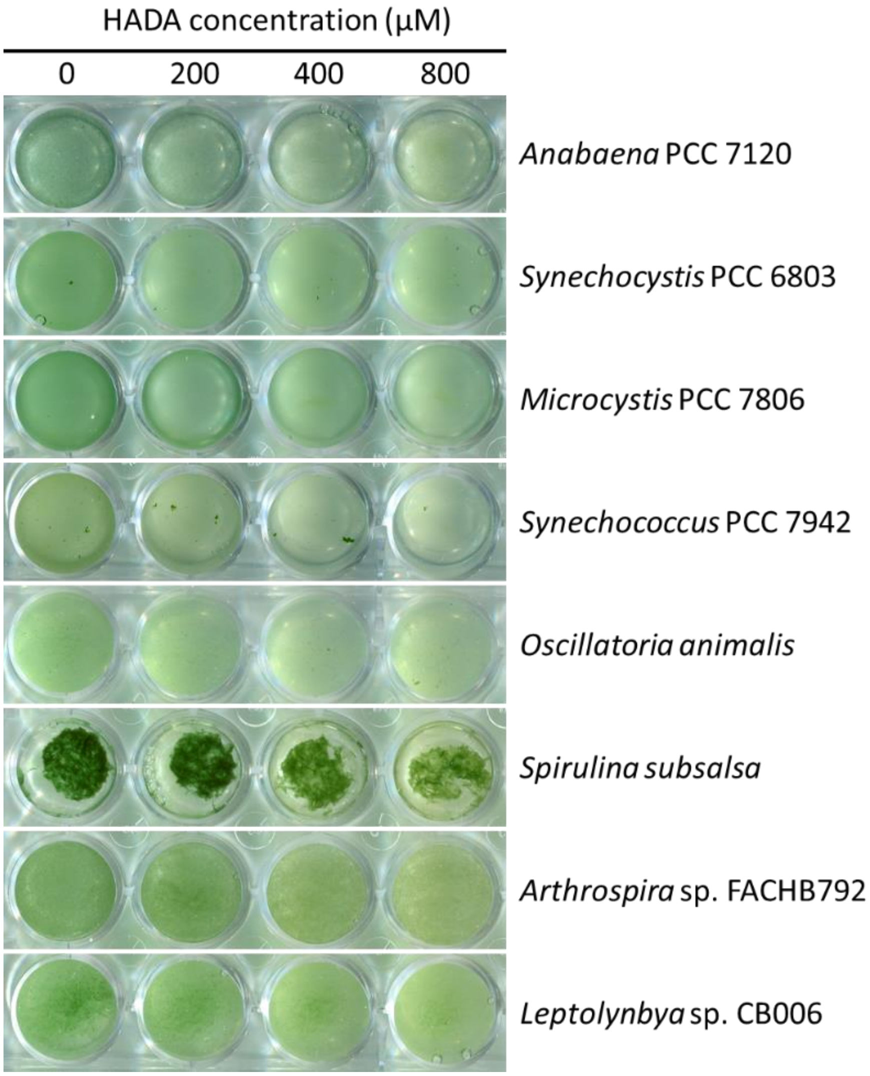

Supplementary Figure 5. The effect of HADA on the growth of the cyanobacterial strains used in this study. Cells were inoculated in 24-well plates in their optimal media (1 ml per well) containing the indicated concentrations of HADA to OD_{750} = 0.15, and grown under the light density of 30 μmol $m^{-2}s^{-1}$ at 30 °C. The plates were imaged after 4 days of growth.
